# Adverse Childhood Experiences of Urban and Rural Preschool Children in Poverty

**DOI:** 10.3390/ijerph16142623

**Published:** 2019-07-23

**Authors:** Leanne Whiteside-Mansell, Lorraine McKelvey, Jennifer Saccente, James P. Selig

**Affiliations:** 1 Department of Family and Preventive Medicine, University of Arkansas for Medical Sciences, Little Rock, AR 72205, USA; 2 College of Medicine, University of Arkansas for Medical Sciences, Little Rock, AR 72205, USA; 3 Department of Biostatistics, University of Arkansas for Medical Sciences, Little Rock, AR 72205, USA

**Keywords:** rural health, adverse childhood consequences, poverty, preschool children

## Abstract

Adverse childhood experiences (ACEs) have long-term health consequences. Young children in the southern part of the United States (US) are at greater risk than children in other parts of the US. This study assessed preschool children ACEs using a family-friendly tool, the Family Map (FMI), and compared children living in rural/urban areas while examining the potential moderation of race. The FMI–ACE score was examined as a total and two sub-scores. We found that race did not moderate the FMI–ACE score but that Black children (Cohen’s d = 0.52) and children in urban and large rural areas were at highest risk (Cohen’s d = 0.38). However, the subscale FMI–ACEs parenting risk was moderated by race such as that Black children were less at risk in rural areas than urban (Cohen’s d = 0.62). For FMI–ACEs environmental risk, race moderated risk such that Black children were most at risk in large rural areas but less so in small rural areas (Cohen’s d = 0.21). Hispanic children were most at risk in small rural areas and least in large rural environments. Findings from this study suggest that targeting the most at-risk children for interventions should consider the context including race and location.

## 1. Introduction

Children living in rural communities in the United States (US) are at greater risk than children in urban areas for an array of health and developmental conditions including obesity and unintended injury [1,2]. Some of the contributors to these serious health conditions experienced by rural children are known. For example, many children in rural America live in poverty and children living in poverty are more likely to have developmental concerns [2,3,4,5]. Rural parents more often have less education and less access to consistent full-time employment than urban parents—both linked to health outcomes [6]. While the challenges experienced by those in rural areas are far from homogeneous, remoteness is a factor in access to health services, including preventive healthcare services and trained health and education staff [3,7].

However, research on the environmental experiences of children has led to a new contributor to health inequities, adverse childhood experiences (ACEs). ACEs are traumatic events experienced before the age of 18 years. While studies have used different measures of ACEs, constructs typically assessed include the direct experiences of maltreatment (physical, sexual, or emotional abuse and/or physical or emotional neglect) and family and parent characteristics [8]. The latter are associated with family stress or correlates of maltreatment, such as instability and disruptions in the family (incarceration of a family member, domestic violence, parental separation/divorce) or characteristic of a parent (mental illness, substance abuse). The 1998 seminal study by Felitti and colleagues linked these 10 indicators of ACEs (Kaiser-ACEs) with adult health consequences [9]. Their study provided the rationale for the 10 adverse events and has guided subsequent research. In the intervening years, a range of studies confirmed and extended the evidence of negative physical and psychological consequences [10]. Combined, these studies document that experiencing even one adversity increases the risk of negative outcomes [11,12,13,14,15].

Recent national estimates indicate that as many as 60% of adults in the US experienced at least one ACE as children [16]. A key finding by Felitti and colleagues is a consistent linear (dose-response from zero to 10) relationship between the number of adverse events experienced and the likelihood of negative consequences. About 25% of adults are thought to have experienced three or more ACES and 9% report five or more [16]. There are many examples of the cumulative effect of ACES on developmental outcomes. For example, compared to adults with no ACEs, those with five or more ACEs had a 2.6 times greater risk of chronic obstructive pulmonary disease (COPD) [17]. This cumulative effect of the number of ACEs has been shown repeatedly across a wide range of physical and mental health outcomes [18].

While the percentage of the general population experiencing at least one ACE is high, nearly all of the studies are retrospective in adult populations. The most nationally representative data on ACEs in the lives of children (not adults retrospectively reporting about their childhood) comes from the National Survey of Children’s Health (NSCH) [2]. However, this study is limited because of differences in the assessment of ACEs from the assessment of ACEs in the original Kaiser study. The NSCH-ACEs measure does not assess indicators of child maltreatment. This may be because asking parents directly about abuse and neglect is problematic. The NSCH-ACEs measure included household dysfunction indicators similar to the Kaiser-ACEs studies. The NSCH-ACEs included measures of parental death, racial discrimination, witnessing neighborhood violence, and family economic hardship in the cumulative ACE score. While there is a rationale for these shifts in definition, it makes an investigation of differences by race and location (e.g., urban, rural) difficult. 

The NSCH documented differences by geographic area, with 53% of children (birth to 18 years) living in small rural areas having had at least one ACE (as defined by NSCH), compared to 47% of children living in urban areas [2]. The NSCH suggested that the disparity of risks begins early. In NSCH, among children between birth and 5 years, 41% of rural children compared to 35% of children in urban areas had already experienced one ACE. Still, the geographic pattern is not that simple. The NSCH defined three geographic areas: urban, large rural (populations of 10,000 to 49,999), and small rural (populations of 2500 to 9999 persons and their surrounding areas) [1]. Across children of all ages, more living in large rural areas (45% for children birth to 5) experienced at least once ACE compared to children in the other two geographic areas. 

Although the NSCH-ACE indicator is confounded by poverty status and racial discrimination, other studies also suggest the prevalence of ACEs differs by geographic area and/or racial groups [3,19]. In general, rural and southern states have higher rates than more urban and coastal states [20,21]. Racial differences in ACE exposure have been documented in multiple studies including adults and children, such as the NSCH. In the original ACEs study by Felitti and colleagues, participants who identified as Black, Hispanic, or “other” reported higher exposure to two, three, or four ACEs compared to white participants [9]. Focusing specifically on abuse, in a recent cohort study of 5502 participants, Black women reported higher rates of physical abuse in their childhoods than white women, whereas white women reported more emotional abuse in their childhoods than Black women. The results were slightly different for men, with white men reporting higher rates of emotional and sexual abuse and Black men reporting more physical abuse [22].

There is evidence that ACEs representing family instability, such as parental separation, domestic violence, and incarceration of a household member, vary along racial and geographic lines. Parental separation/divorce is the most common ACE and is known to be associated with poorer academic outcomes and interpersonal skills for children during the period of the divorce [23,24]. A recent study of the NSCH data found that US-born Hispanic children are more likely to experience parental separation/divorce than white, Black or immigrant Hispanic children [25]. Black and Hispanic children are more likely to be exposed to parental incarceration and domestic violence between parents compared to white children [25,26]. In the context of geographic setting, a cross-sectional analysis of children in South Carolina found slightly reduced rates of parental separation, domestic violence, and incarceration of a household member among rural participants [23]. Overall, minority children experience parental separation, domestic violence, and household incarceration more often than white children do. Domestic violence is more frequent in rural areas [27,28]. 

Other ACE indicators that threaten the optimal development of children within the household differ by race and geographic location. For example, there are racial and geographic disparities in substance abuse and mental illness. While illicit drug use and alcohol abuse are reported at approximately equal rates among urban and rural residents ages 12 and up, the risk for many mental illnesses, such as mood, psychotic, and anxiety disorders, is higher in cities [29,30]. For example, the risk of schizophrenia shows a dose-response relationship with time spent in a city during childhood [30]. When examined in the context of race, Hispanic, Black, and white children of US-born parents are approximately equally likely to have a household member with mental illness [25]. However, Hispanic children are more likely than white children to be exposed to household drug or alcohol abuse, putting them at increased risk of other ACEs such as abuse or neglect [25]. 

The assessment of current childhood adversity and the ability to examine links is a serious gap in the current research foundation. The research findings to-date are lacking in several areas. As mentioned, the NSCH survey’s modified assessment of ACEs excluded indicators of childhood abuse and neglect. Examining this distribution is key to developing effective interventions to prevent the negative physical and psychological outcomes associated with ACE exposure [9,31]. Further, NSCH used poverty and the experience of racial discrimination as indicators of adverse experiences of children. This may be related to the conflictual findings between children and adults in the prevalence of rural-urban ACES. That is, while the NSCH suggests that children living in rural locations are more at risk; studies of adults suggest that adults in urban areas report a higher history of risk from childhood experiences [23,32]. While these changes to the definition of an ACE indicator may be justified, it makes it difficult to understand the different experiences of children as defined by Felitti and colleagues [9]. This inclusion makes the task of researchers and policy-makers focused on the levels of risk within the population of children living in poverty more difficult.

Our study expanded on the 2012 portrait of rural children’s experiences described by the National Survey of Children’s Health (NSCH) by focusing on a large sample of children living in poverty in a Southern state [33]. We focused on a sample of preschool children enrolled in early childhood education (ECE) programs (both center- and home-based programs) serving low-income families. The sample was diverse in race and geographic location. We used location designations similar to the NSCH. We used a definition of childhood adversity that was constructed to align with the Kaiser-ACEs while assessing the experiences of preschool children [15]. The validated survey tool was developed to support family engagement in ECE programs serving children ages 3 to 5 years [34]. 

Analyses to date have documented race/ethnicity and geographic location as risk indicators but not the potential moderation of one on the other. In this study, we examined the combination of race and geographic location, both of which are linked to adversity for children and families. In all, our study was the first to examine ACEs for children specifically in the preschool years while examining the interaction of race and geographic location on adversity exposures. 

## 2. Materials and Methods

### 2.1. Participants

Participants (N = 3911) were parents with preschool-aged children receiving early childhood services in Arkansas. Participants were a convenience sample of parents and/or children enrolled in programs serving low-income families. Programs served children from 3- to 5-years-of-age in center-based childhood education or home-based parenting education programs. Six Head Start agencies provided data from 2028 families [35]. Head Start programs served children with income not exceeding 100% of poverty or other at-risk criteria (children in foster care or with developmental delay/disability) [35]. Four state-funded pre-Kindergarten programs provided data from 554 participants. State-funded programs served children with income not exceeding 200% of poverty or other at-risk criteria (adolescent parent, limited English proficiency, parent history of substance addiction or abuse/neglect) [36]. Fifteen Home Instruction for Parents of Preschool Youngsters (HIPPY) home visiting programs provided data for 1309 families. HIPPY’s goal is to promote school readiness with age-appropriate parent teaching in the home. Families were eligible for HIPPY based on demographic characteristics (low-income or a single and/or teen parent), parent characteristics (such as parental history of abuse, incarceration, military deployment, disability, or chronic illness), and child characteristics (developmental delay, pre-term/low birth-weight, disability, or chronic illness) [15]. The Institutional Review Board of the University of Arkansas for Medical Sciences approved the study (Protocol #133672).

### 2.2. Procedures

Data for this study focused on the Family Map interview (FMI) conducted by program staff during enrollment or soon after. Data were extracted from program records and scanned into an electronic system. Children’s race and ethnicity were reported by caregivers during the interview. Participants with missing information for race/ethnicity that were not from one of the target races (i.e., white or Black) or ethnicity codes (i.e., Hispanic) were excluded (n = 239; n = 132: Head Start; n = 33: State-funded; n = 74: Home Visiting). Further, 20 participants from one Head Start agency were excluded because their location code was missing. The resulting analyses data set included 3652 participants.

Over 180 trained program staff interviewed the primary caregiver (e.g., parent) using The Family Map Inventory-Early Childhood (FMI-EC) [34,37]. All program staff conducting interviews received 6 hours of training. On-site support in the administration of the interview was provided as needed by the research team. Most caregivers interviewed were the biological mother (86.4%) or father (5.7%). Other caregivers interviewed were the child’s grandmother (4.2%), a step or adoptive parent (1.9%), other relative (0.4%), or other adult (foster parent, 0.7%, unknown, 0.7%). Interviews were collected within the first 3 months of the child’s enrollment in the program. Data were extracted in cohorts over multiple years such that no child could be represented more than once (from 2014 to 2016). The county of the agency was coded for each family and linked to 2013 Rural–Urban Continuum Codes to classify the community by population size and location to another metro area with up to nine codes [38].

### 2.3. Instruments

The Family Map Inventories (FMIs) were designed for use by early childhood staff (e.g., ECE/parent educators), the FMIs consist of semi-structured interviews and observation items to assess family and home environment characteristics associated with children’s well-being. There are three versions of FMI based on the child’s age (i.e., prenatal, under 3 years, and 3–5 years) each in English and Spanish [34,37]. This study used the FMI for Early Childhood (FMI-EC, for children 3–5 years-of-age). Using FMI, program staff systemically evaluate risk factors (e.g., parental mental illness, harsh parenting style, food insecurity) and areas of strength (e.g., safe home environment, available learning materials) in families with young children. Beyond identifying service needs, the goal of the FMI is also to support strong parent–teacher partnerships and effective interventions. 

FMI-EC interviews take approximately one hour and consist of 12 domains in three areas: (a) child physical and social experiences; (b) family climate; and (c) parent characteristics. Previous research describes the rationale for each of the 12 constructs, all of which play key roles in healthy child development [34,37]. The FMIs have been shown to have sufficient internal consistency reliability across domains (Cronbach’s alpha range = 0.68–0.90) and test-retest reliability of concordance of risk identification (63–100%) [34,37]. Risks are defined by research-based guidelines (e.g., American Academy of Pediatrics). The FMIs and other national studies find similar rates of family risks in low-income populations [34,37,39,40]. The FMIs have been successfully implemented in home visiting programs, early childhood education programs, including Early Head Start, Head Start, and school- and community-based programs [37]. 

Adverse Childhood Experiences (FMI-ACEs). Specific FMI-EC items (see Table 2) have been shown to be close correlates for the retrospectively-reported Kaiser-ACEs [9,15,31]. Paralleling the Kaiser-ACEs, documentation of any risks within a construct represented a positive screening. However, rather than providing a comprehensive history of risk exposure, FMI-ACEs screens the child’s current environment, permitting the ability to provide appropriate referrals and supports as well as documenting change in risk over time [15]. This method of screening for ACEs has been shown to accurately predict child-maltreatment risk [15]. 

In this study, we examined the total FMI-ACEs score and two sub-scores. The first sub-score represented risks associated with parenting and was composed of the first five risk areas as seen in Table 2 (FMI-ACEs Parenting). The second sub-score represented risks associated with the family and home environment and combined the second set of five items in Table 2 (FMI-ACEs Family). These sub-scores were not used by McKelvey et al.; however, they were consistent with our intent to create and investigate a construct similar to the one used in the NSCH study (i.e., environmental threats) and examine FMI-ACEs Parenting risk separately [15]. Further, a range of research has supported the clustering of ACEs [14,41].

### 2.4. Approach to Analysis

Preliminary analyses examined distributional assumptions and bivariate comparisons of demographic characteristics using χ^2^ tests. Comparisons were conducted (SPSS Version 23.0; IBM, 2015) [38] using ANOVA or logistic regression with main effects and interaction terms (Table 1). ANOVA or logistic regression with post hoc Bonferroni-corrected tests using the Holm–Bonferroni method, which is more powerful than Bonferroni-corrected tests [42,43]. ANOVA was used with caregiver age (in years), caregiver employment (in hours/week), number of adults living in the home, and number of children living in the home. Logistics regression was used to examine differences in child gender, child age (over 54 months), who was interviewed (biological mother vs other), partner in the home, and caregiver education completed (more than high school degree vs less). Holm–Bonferroni-corrected tests examined interactions based on nine tests. When interaction terms were not significant, main effects were examined.

Full Information Maximum Likelihood (FIML) was used to estimate regression models predicting the ACE scores [44,45]. FIML uses all available data and provides the least biased estimates in multiple regression models with missing data compared to other commonly used methods such as pairwise or listwise deletion [46]. FIML estimates were computed using SAS (9.4) [47]. Demographics were included as covariates in multivariate analyses. These included child characteristics (child gender) and family/parent characteristics (parent age, education, employment status, number of children in the home). The results of the FIML tests are shown in Table 2.

## 3. Results

### 3.1. Preliminary Analysis of Racial and Geographic Description of Sample

Children (n = 3652) represented counties from eight of the nine metro and non-metro categories. Category definition was based on the definition from the Office of Management and Budget. In this study, we combined areas into three similar to the NSCH study. The majority of children (65.7%) were in metro areas considered urban. Urban area definition was based on population and worker commuting criteria from census data and includes counties with more than 250,000 people but can contain less. Of the children in non-metro areas, 8.5% were in large rural areas. These areas are counties of populations of 10,000 to 49,999 persons and their surrounding areas. Finally, 25.8% of children were in smaller rural areas. These areas were small towns with populations of 2500 to 9999 persons and their surrounding areas. 

Families were not distributed evenly by race across geographic areas. More Black families lived in urban areas compared to other areas (86.9% in urban, 9.2% in large rural, and 3.9% in small rural, (χ^2^ (4, *N* = 3652) = 888.04, *p* < 0.001). The distribution for white and Hispanic families was more equal with the exception of large rural (white: 41.45, 8.4%, 50.2%; Hispanic: 70.4%, 6.8%, 22.8%, for urban, large rural, and small rural respectively). Immigration status and country of origin were not available for participants; however, based on parent report, more Hispanic parents were comfortable communicating in English in large rural areas (82% compared to 48%) than those in urban or small rural area (χ^2^ (2, *N* = 615) = 16.08, *p* < 0.001).

An examination of demographic characteristics across location and race/ethnicity of participants is shown in Table 1. Two significant interactions (race by location) were identified: number of children in the home (F(4, 3230) = 4.89, *p* < 0.001) and caregiver education levels (Wald χ^2^ (4, *N* = 3300) = 16.39, *p* < 0.003). Black families in small rural areas had fewer children than families of other races/ethnicities in small rural areas and fewer than Black families in urban or large rural areas. On the other hand, Hispanic families in urban areas had more children than other Hispanic families and other urban families. White caregivers in rural locations were 1.9 times more likely than caregivers in urban locations to have more than a high school education (Wald χ^2^ (2, *N* = 3300) = 34.11, *p* < 0.000). However, Black caregivers in rural locations were significantly less likely to have more than a high school education (OR = 0.62, Wald χ^2^(1, *N* = 3300) = 11.70, *p* < 0.001).

Where interactions of race and location were not significant, differences in demographics based on the main effects of race and location were examined. Race/ethnicity differences were found in homes in which the caregiver had a partner, number of adults in the home, and employment. Regardless of location, only about 40% of Black families had two caregivers in the home compared to about 70% of families of other races/ethnicities (Wald χ^2^ (2, *N* = 3652) = 84397, *p* < 0.001). Black caregivers reported working more hours per week than other participants regardless of location (F(2, 3372) = 7.36, *p* < 0.001). For example, 58.4% of Black caregivers worked 21 or more hours a week compared to 44.5% for Hispanic caregivers and 45.6% for white caregivers. All families in large rural areas had fewer adults in the home than families in other locations (F(2, 3365) = 7.03, *p* < 0.001). Among Hispanic families, the number of adults in the home varied with the smallest families living in large rural areas and those with the most household size in urban areas (F(2, 3365) = 12.15, *p* < 0.001).

### 3.2. Total FMI-ACE Score by Race/Ethnicity and Geographic Location

One-third of children experienced no ACEs (33.3%). However, most (66.6%) of the children in our sample had experienced at least one adverse event, with 28.6% experiencing one, 19.5% experiencing two, 10.1% with three, 4.8% with four, and 3.7% experienced more than four. As seen in Table 2, the average FMI-ACE score was 1.38 (SD = 1.42, range 0-8). The FMI-ACEs Parenting score was 0.74 (SD = 0.91) and the FMI-ACEs Family score was 0.63 (SD = 0.81).

In preliminary analyses, we examined the bivariate association of ACE scores across the locations and race/ethnicity of participants. Bivariate ANOVA indicated that the total FMI-ACE score differed by location (F(2, 3413) = 33.69, *p* < 0.001) with post hoc, Holm–Bonferroni -corrected tests indicating that urban and large rural areas had higher FMI-ACE scores (M = 1.50, SD = 1.45, M = 1.47, SD = 1.33, respectively) than small rural areas (M= 1.05, SD = 1.30). This pattern is confirmed with an examination of the percent of children with four or more FMI-ACE by location: Urban 9.7%, Large Rural 7.8% and Rural 5.7%.

Similar ANOVA analysis found differences by race/ethnicity (F(2, 3413) = 49.60, *p* < 0.001) with Black children having the highest FMI-ACE score (M = 1.60, SD = 1.43) followed by white children (M = 1.25, SD =1.33) and Hispanic children (M = 1.01, SD = 1.34). This pattern is confirmed with an examination of the percent of children with FMI-ACE score at or above 4 by race: Black 10.4%, white 7.8% and Hispanic 5.5%.

Next, we examined the hypothesis that the total FMI-ACE score differed by location and ethnicity (i.e., interaction) using multivariate analyses. Missing data ranged from 11% (e.g., the number of children in the home) to less than 1% (e.g., relationship of child to caregiver). An examination of missing data indicated that the use of listwise deletion would result in a significant loss of cases, thus SAS/FIML was used in multivariate models to estimate FMI-ACE scores. Based on this model, after controlling for family, child characteristics, the main effects of race/ethnicity, and location, we entered four interaction terms in the model. No interaction term was significant.

An examination of the main effects in the SAS/FIML multivariate model without interactions found similar results as the bivariate analyses for race/ethnicity with Black children having higher FMI-ACE scores than whites and Hispanic children having lower FMI-ACE scores than whites (t(1) = 2.12, *p* = *0*.04; t(1) = –5.38, *p* < 0.001). The Cohen’s d effect size between the number of FMI-ACEs between Black and white children was moderate in size at 0.52. Across geographic locations, differences were identified between children in rural areas. Based on this analysis, children in rural areas had lowest total FMI-ACE score but children from urban and large rural areas had similar FMI-ACE score (t(1) = –3.31, *p* = *0*.00; t(1) = –0.33, *p* < *0*.74). The Cohen’s d effect size between the number of FMI-ACEs between urban and rural children was smaller at 0.38.

### 3.3. FMI-ACEs Parenting and Family Sub-Scores

For FMI-ACEs Parenting, our analyses indicated that race moderated the risk score across locations. That is, in urban and large rural areas, a consistent pattern of FMI-ACEs Parenting risk was seen with Black children at highest risk (M = 0.92, M = 0.83), white children at moderate risk (M = 0.69, M = 0.72) and Hispanic children at lowest risk (M = 0.56, M = 0.37). However, in rural areas, white children (M = 0.61) were at the highest risk and Black children at lowest risk (M = 0.46, t(1)= –2.69, *p* < 0.007). The Cohen’s d effect size between the number of FMI-ACEs Parenting risks between urban and rural Black children was moderate at 0.62. Hispanic children’s risk was not statistically different across locations.

For FMI-ACEs Family, our analyses indicated two significant interactions with shifts between the three geographic locations. Children from white families (M = 0.94, 0.93, 0.86 for urban, large rural, small rural respectively) had relatively stable risk across location. Children from Black families living in large rural areas were more at risk than small rural areas (M = 0.87, 0.97, 0.77 for urban, large rural, rural respectively). The Cohen’s d effect size between the number of FMI-ACEs Family risks between small rural and large rural Black children was smaller at 0.21. However, children from Hispanic homes (M = 0.74, 0.65, 0.83 for urban, large rural, small rural respectively) had significantly low risks in large rural areas compared to other children. For example, the effect size between white and Hispanic families living in large rural areas was 0.31.

## 4. Discussion

Our study provided the first investigations of exposure to ACEs across geographic setting considering the potential for race/ethnicity to act as a moderator within the context of poverty [33]. In a range of studies, ACEs have been linked to long-term adult health and wellness. The concern for children in rural areas is twofold. First, the NSCH suggested that rural children are at greater risk for adverse experiences. Second, children and their families living in poverty within rural communities have unique challenges. Our intent was to expand on the findings of NSCH by examining ACEs using a definition more closely aligned with the Kaiser-ACEs definition in the context of poverty in the rural south.

Beyond the value of our study to examine the moderation of risk, the study is valuable as one of the few studies of geographic comparisons for ACEs in young children. In fact, the Family Map Inventories are one of few tools with widespread use targeting very young children [8]. There have been a few studies of ACEs in rural areas using the Centers for Disease Control and Prevention’s (CDC) Behavioral Risk Factor Surveillance System (BRFSS) in adults [18]. A review of these results suggests that rural and urban adults reporting on past ACEs appear to have roughly the same levels of risk regardless of current residence [48]. However, the assessment of adult reports of childhood experiences at least 18 years ago is less helpful for targeting efforts to address and prevent ACEs for young children.

Findings from our study suggested that an examination of the total NSCH-ACE score does not provide an accurate summary of the risk exposure for low-income children and when comparing rural children to urban children. In the NSCH study, racial experiences were assessed with a question of experienced racial discrimination. We approached this differently, in part because the children in our study were young. Based on our analyses of the total FMI-ACE score, race was not a moderator of risk. However, contrary to the NSCH study, children living in rural areas were less often exposed to adverse events than children in urban areas regardless of race. Still, Black children experienced more FMI-ACEs than other children regardless of location.

We examined two sub-scores intended to assess the exposure of children to risks associated with parenting and family demographic risk factors separately. When the risks related to FMI-ACEs Parenting were examined, the level of risk across geographic locations for adverse childhood experiences depended on race. That is, an examination of the number of FMI-ACEs Parenting score suggested that urban areas were associated with more risk (e.g., 0.81 urban vs 0.55 rural risks). For example, children from Black families were at higher risk in more populated areas but at less risk for parenting-related risks associated with child maltreatment in rural areas. However, the risk is relatively level for white and Hispanic families, which have less risk in large rural areas compared to urban areas. The reduction of risk in rural areas for Black and Hispanic families compared to urban areas was such that white children had the highest risk in rural areas.

Overall, results from FMI-ACEs Family risks were similar to FMI-ACEs Parenting in that rural areas present less risk to children than urban areas for many children. This subscale is somewhat similar to the one used in the NSCH study. However, the studies differed in where children most at risk lived. In the NSCH study, rural children were more at risk than urban children. In our study, risk was dependent on the geographic location of the child. That is, for the FMI-ACEs Family scale, Hispanic children living in large rural areas had more risks than rural Hispanic children; but the situation was reversed for Black children. Black children in rural areas had the fewest risks and black children in large rural areas the most. This finding may be a function of the difference in the way each study treated racial influences. In our study we compared the risks by racial groups whereas the NSCH study included the experiences of racial discrimination as part of the risk experienced.

One limitation, but also potential strength, of our study is the economic homogeneity of the study population, since all of the children were from low-income families. The ACE prevalence found in our study participants is likely to be higher than the general population, as children living in poverty are more susceptible to ACE exposure [19]. Future research should replicate our study in a more economically diverse population to determine if the patterns of specific ACE exposure remain consistent across varying socioeconomic statuses. However, our focus on children from low-income families is also a strength as it guides our understanding of the children who are at highest risk of ACEs and their negative consequences, allowing for the development of more precise and effective interventions for the children who need it most.

Another strength of the study is examining ACEs using the FMI. The FMI-ACEs represent proxies for ACEs (e.g., asking if the parent spanks with objects, rather than asking about confirmed physical abuse) that are measured within a specific period. This is particularly relevant as asking parents directly about ACEs could be potentially incriminating (e.g., ACEs represent stigmatized and potentially illegal activities), making them likely unreliable. Further, the FMI-ACEs screening provides an avenue for clinicians and interventionists to identify risks while maintaining a working relationship with parents, whose engagement and eventual behavior modification are critical for reducing the identified risks.

## 5. Conclusions

Overall, this study provides a new perspective on ACEs and the children who experience them by examining ACE exposure in terms of both geography and race. Retrospective studies of adults, while helpful, are not as useful in directing resources and intervention efforts as studies of the current experiences of children. By taking into account urban versus rural environment and white versus minority status, we were able to examine each group’s risk factors pertaining to parenting and their home environment. Our study provided strong evidence that individual ACEs are not experienced equally across racial and geographic divides. This information has public health implications, as it is critical to know which children are most at risk for specific ACEs in order to implement proper screening. As we learn more about ACE exposure and its consequences, the need for ACE screening in pediatric clinics has become apparent. More work is needed to establish the feasibility and acceptability of screening for these sensitive topics in the clinical setting; however, there is already some evidence showing that ACE assessment can be viewed positively by parents [49]. Furthermore, the next step after screening is to develop interventions that are culturally appropriate and can be practically implemented to assist families in ameliorating their unique risk factors. Currently, there is evidence to support interventions such as parenting education programs and mental health treatment. It would be beneficial to identify in future work when interventions are equally effective across racial and geographical differences [50,51,52].

## Figures and Tables

**Table 1:** Comparisons of characteristics across location and race/ethnicity of participants.

	Urban			Large Rural			Rural			
	White	Black	Hispanic	White	Black	Hispanic	White	Black	Hispanic	Total
N	620	1377	402	125	146	39	751	62	130	3652
Percent	17.0%	37.7%	11.0%	3.4%	4.0%	1.1%	20.6%	1.7%	3.6%	100%
Child Male ^b^	46.6%	48.7%	50.9%	52.9%	52.7%	47.2%	54.1%	44.8%	54.3%	50.2%
Child Age ^b^										
42 months or less	26.9%	29.6%	27.8%	34.7%	35.3%	43.2%	33.1%	21.1%	26.6%	30.0%
43–54 months	46.8%	46.7%	44.8%	45.2%	39.7%	37.8%	44.4%	43.9%	48.4%	45.6%
> 54 months	26.3%	23.7%	27.5%	20.2%	25.0%	18.9%	22.5%	35.1%	25.0%	24.4%
Caregiver Interviewed										
Biological Mother ^b^	78.3%	81.7%	82.3%	84.8%	80.4%	94.9%	84.5%	82.3%	82.9%	82.0%
Biological Father	6.9%	4.8%	7.1%	5.6%	2.8%	2.6%	4.4%	4.8%	10.1%	5.4%
Other	14.9%	13.5%	10.6%	9.6%	16.8%	2.6%	11.0%	12.9%	7.0%	12.5%
Partner in Home **^,b,d^	70.8%	40.2%	72.9%	60.0%	41.1%	59.0%	76.4%	46.8%	76.2%	58.7%
Caregiver Age ^a^										
24 years or less	22.3%	21.7%	16.8%	24.5%	22.7%	28.1%	19.5%	27.1%	17.2%	20.9%
25–34 years	52.6%	58.8%	56.4%	55.9%	57.6%	62.5%	58.5%	52.5%	51.7%	56.9%
35 or older	25.1%	19.4%	26.7%	19.6%	19.7%	9.4%	22.0%	20.3%	31.0%	22.1%
Employment *^,a,d^										
Not Working	47.8%	31.9%	42.6%	43.0%	35.6%	28.1%	40.3%	30.5%	47.4%	38.6%
20 hours or less	10.4%	9.7%	13.1%	15.0%	6.7%	9.4%	10.4%	10.2%	12.1%	10.5%
>20 hours	41.8%	58.4%	44.2%	42.0%	57.8%	62.5%	49.3%	59.3%	40.5%	51.0%
Education *^,b,c^										
No Degree	11.2%	8.1%	46.0%	20.4%	8.2%	37.0%	5.5%	10.2%	43.1%	14.2%
High School Degree	54.4%	54.5%	45.2%	49.0%	50.8%	44.4%	45.1%	62.7%	39.7%	50.7%
Some College	34.4%	37.4%	8.9%	30.6%	41.0%	18.5%	49.4%	27.1%	17.2%	35.1%
Adults in Household **^a,e,d^										
Mean	1.17	0.90	1.42	1.09	1.06	0.79	1.04	0.93	1.06	1.11
SD	0.89	0.85	1.00	.81	0.95	0.74	0.88	0.77	0.70	0.88
Children in Household **^,a,c^										
Mean	1.61	1.66	1.77	1.45	1.49	1.70	1.38	1.38	1.78	1.50
SD	1.23	1.36	1.25	1.18	1.45	.99	1.08	1.32	1.33	1.17

* *p* < *0*.05, ** *p* < *0*.01 with ^a^ ANOVA or ^b^ Logistic Regression including main effects and interaction terms (race by geographic area) followed by Holm–Bonferroni method corrections for number of tests. ^c^ Significant interactions; ^d^ Significant main effects race, ^e^ Significant main effects location

**Table 2:** Adverse childhood experiences from family map inventories with unadjusted percent and means.

Original Ace	Family Map Item	Race/Ethnicity			Geographic Location			Total
Construct	Description	White	Black	Hispanic	Urban	Large Rural	Rural	
FMI-ACEs-Parenting Subscore ^a^ **	**Mean (item 1–5)**	0.64	0.88	0.60	0.81	0.74	0.55	0.74
	SD	0.87	0.94	0.86	0.93	0.83	0.84	0.91
1. Emotional Abuse	Family Members Verbal Anger/Discipline	5%	8%	5%	7%	4%	5%	6%
2. Physical Abuse	Exposure to Violence or Physical Discipline	19%	33%	13%	26%	26%	18%	24%
3. Sexual Abuse	Child Exposed to Sexual or Open Child Protective Services	6%	4%	2%	5%	4%	5%	5%
4. Emotional Neglect	Family Members Not Cohesive	12%	22%	18%	21%	15%	8%	17%
5. Physical Neglect	Food Insecurity, Homeless or Crowded Home	22%	20%	21%	22%	21%	19%	21%
FMI-ACEs-Family Subscore ^a,^**	**Mean (item 6–10)**	0.59	0.76	0.41	0.68	0.75	0.49	0.63
	SD	0.82	0.81	0.72	0.82	0.85	0.76	0.81
6. Parental Separation or Divorce	Parent Living Outside the Home and/or Owed Child Support	32%	51%	23%	42%	49%	27%	39%
7. Mother Treated Violently	Someone in Home Hurt	6%	6%	4%	7%	7%	3%	6%
8. Household Substance Abuse	Friends/Family with Drinking/Drug	2%	2%	3%	2%	1%	2%	2%
9. Household Mental Illness	Depression Screen	10%	7%	7%	8%	7%	9%	8%
10. Incarcerated Household Member	Family Involved w/Legal System	11%	9%	4%	9%	10%	8%	9%
FMI-ACEs-Total Score **^a^**	Mean (items 1–10)	1.25	1.63 **	1.01	1.50	1.47	1.05 **	1.38
	SD	1.38	1.43	1.34	1.45	1.33	1.30	1.42
Number of Aces								
0		38.3%	23.3%	47.6%	29.4%	28.1%	44.6%	33.3%
1		27.8%	30.2%	26.3%	28.7%	27.0%	28.8%	28.6%
2		17.0%	23.7%	14.5%	21.0%	26.7%	13.5%	19.5%
3		9.1%	12.5%	6.2%	11.1%	10.4%	7.5%	10.1%
4+		7.8%	10.4%	5.5%	9.7%	7.8%	5.7%	8.5%

**p < 0.01 ^a^ Comparisons controlled for child (age, gender), parent (age, education, work status), and family (number of children in home) characteristics and adjusted for missing data with FMIL; however values in table are unadjusted.
